# Exercise-Induced Bronchospasm in Elite Athletes

**DOI:** 10.7759/cureus.20898

**Published:** 2022-01-03

**Authors:** Konstantinos M Pigakis, Vasileios T Stavrou, Ioannis Pantazopoulos, Zoe Daniil, Aggeliki K Kontopodi, Konstantinos Gourgoulianis

**Affiliations:** 1 Respiratory Medicine, Creta Interclinic Hospital, Heraklion, GRC; 2 Pulmonology, University of Thessaly, Larissa, GRC; 3 Emergency Medicine, University of Thessaly, Larissa, GRC

**Keywords:** cardiopulmonary exercise test, airway dehydration, airway hydration, elite athletes, athletic asthma, exercise induced bronchoconstriction, exercise induced bronchospasm

## Abstract

Exercise is one of the most common triggers of exercise-induced bronchospasm (EIB), with less trained athletes showing more symptoms. Exercise-induced bronchospasm is a common and frequent problem among elite athletes with obvious implications on competing performance, health, and quality of life. Classical pathways in the development of EIB in this population include the osmotic and the thermal theory as well as the presence of epithelial injury and inflammation in the airway. Moreover, neuronic stimulation has been suggested as a potential modulator of EIB in elite athletes. In this category of population, the diagnosis of EIB is a serious challenge, especially as spirometry before and after bronchodilation is not diagnostic and specific tests are required. To date, there is no organized screening in asymptomatic elite athletes to detect EIB. This review aims to summarize the pathophysiology, clinical manipulations, and therapeutic approach of EIB in elite athletes. We searched for published studies related to the aim of this study. Exercise-induced bronchospasm is a serious and common disorder in elite athletes, and its symptoms are nonspecific with a need to confirm the diagnosis with specific tests.

## Introduction and background

Exercise-induced bronchospasm is defined as the transient airway contraction after intense exercise without the existence of a medical history of bronchial asthma [1]. Exercise is one of the most common EIB triggers [1,2] and its diagnosis in elite athletes is important due to the potential limitation of their performance [3]. "If from running, gymnastics, or any other work, breathing becomes difficult, it is asthma,” described Greek physician Aretaeus of Cappadocia in 100 A.D. [4,5]. The term EIB began to be used in the 1960s when an article nominated the bronchospasm following exercise in children as “exercise-induced asthma” [5,6]. Now, many specialists support using the terminology 'EIB' instead of exercise asthma as it does not mean that the patients have underlying bronchial asthma [7]. Exercise-induced bronchospasm in athletes has strange clinical characteristics and consequently, some investigators believe that it is a different phenotype [7]. Many elite athletes with EIB have no history of asthma, suggesting that in these cases environmental factors are further significant than genetic factors. So, many specialists proposed to designate EIB with asthma (EIBA) with the appearance of bronchospasm after physical activity in asthmatic athletes, and EIB without asthma (EIBWA) where the appearance of bronchospasm is triggered by exercise in athletes without other symptoms of asthma [7]. Both EIBA and EIBWA share bronchial hyperactivity. Differentiating among them is significant for correct diagnosis and treatment. It has been shown that athletes exercising water and winter sports have a high risk of developing “athletic asthma” corresponding to EIBWA. This fact complies with the assumption of the type of sport and the environmental conditions during exercise and competition that influence the appearance of EIB [7]. For the aims of this review, EIBWA will be referred to as EIB.

Exercise-induced bronchospasm is more common in elite athletes than amateur athletes or people who do not exercise [8,9]. The prevalence of EIB is 7% to 10% of the general population and 20% to 50% of elite athletes, especially those engaged in high-intensity aerobic exercise [10-12]. A study conducted among the USA Winter Olympics athletes found that EIB affected one in every four winter sports athletes and that EIB was more prevalent among women [13]. Various environmental factors can affect a trainee’s performance especially low temperatures, cold air, chlorine products, various irritant pollutants, and air humidity levels [14-18]. The risk of EIB increases in atopic asthma, but not in asthmatic athletes when compared with nonatopic athletes [5]. 

The main stimulus for the appearance of EIB is hyperventilation [19] and the hyperosmotic environment on the airway surface [20-23]. Also, in elite athletes, the extreme ventilation conditions cause airway epithelium injuries with the release of special mediators that trigger EIB [1,24-28].

This review aims to summarize the pathophysiology, the clinical manifestations, the diagnostic manipulations, and the treatment of EIB, as well as to study its possible long-term effects on the lung functions of elite athletes.

We conducted this study by searching the electronic databases of Medical Literature Analysis and Retrieval System Online (MEDLINE), PubMed, Google Scholar, and Scopus for studies published in the English language between the years 1980 and 2021. The following terms and keywords were used to generate the search: exercise-induced bronchospasm, elite athletes, athletic asthma, spirometry in athletes, provocation tests, cardiopulmonary exercise tests. Fifty-five articles were found to be compatible with the aim of this study.

## Review

Pathophysiology of EIB

EIB in elite athletes is the result of years of intensive training, but the exact mechanisms of its development have not been established with certainty [1,2,3]. Intensive exercise leads to the breathing of high volumes of cold and dry air, resulting in key pathogenic events related to EIB such as bronchial osmotic changes, bronchial epithelial damage, bronchial inflammation, and neuronal stimulation [5,7]. During high-level workouts, athletes must push their bodies to their limits. In doing so, they trigger all the systems of the body to work faster and harder. During exercise, athletes can increase their ventilation (VE) up to 20 to 30 times in correlation to rest [19]. Due to hyperventilation, the breathed-in air does not obtain the desired humidity and temperature, resulting in water evaporation and the creation of a hyperosmotic environment on the airway surface. When the levels of VE exceed 30 liters/min, nasal breathing becomes oronasal breathing and the airway is exposed to a greater amount of air that has not been heated, as well as microparticles, droplets, and pollutants such as chloramines and ozone [20-23]. The hyperosmosis leads to the cellular activation and release of special mediators, which cause bronchospasm. The loss of heat caused by hyperventilation is followed by reactive hyperemia, which can contribute to bronchospasm. In elite athletes, the extreme ventilation condition causes mechanical overload and microtrauma to airway epithelium with the release of special mediators that trigger EIB, and may also contribute to airway remodeling [24]. The airway dehydration in high airflow conditions is implicated in the epithelium detachment and its injury, as it has been shown in animal models, and humans [25,26]. There is no consensus on whether EIB is one type of asthma or a distinct disease associated with airway damage due to intense exercise [27]. Some studies suggest that the long-term and high-intensity training in endurance sports may not only stimulate EIB but also stimulate the subsequent appearance of bronchial hyperreactivity outside exercise conditions or permanent airway remodeling [28,29,30]. Although bronchial inflammation reduces after ending competitive sport, the prevalence of asthma diagnosis and use of medication increase in both active and past athletes [5,7].

**Figure 1:**
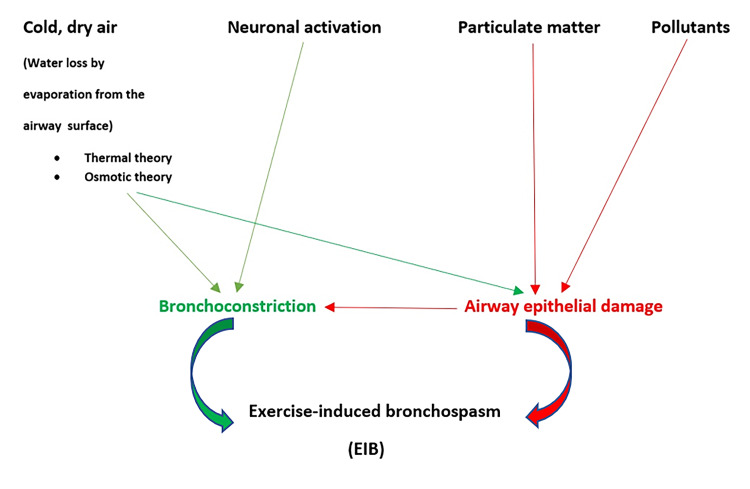
Pathophysiology of EIB in elite athletes EIB: Exercise-induced bronchospasm

Three main theories have been proposed to interpret the mechanism of EIB namely, the thermal theory, the osmotic theory, and the theory of epithelium microtrauma [22,24,31]. The thermal theory supports that the cooling of the bronchial walls because of dehydration, causes local vasoconstriction. Low respiratory tract cooling stimulates cholinergic receptors in the airways, increasing both bronchial smooth muscle tone and bronchial secretions [7]. During re-heating, bronchial stenosis is caused by the mechanical effects of vascular distension, vascular congestion, increased vascular permeability, and bronchial wall edema [10]. In this case, there is no release of mediators or contraction of the bronchial smooth muscle (bronchospasm), but the bronchial stenosis is a direct effect of vascular events (Figure 1) [32]. The osmotic theory supports that the bronchial dehydration that takes place during intense exercise leads to a hyperosmotic environment on airways, which activates cellular mechanisms that lead to the release of various mediators. This results in the contraction of the smooth muscle fibers of the bronchial walls and consequently bronchial stenosis (Figure 1) [33]. However, this hyperosmotic stimulus is present in all athletes, and therefore, all experience bronchospasm during or after exercise. This is not the case, however, with the theory of epithelium microtrauma which directs research to look for other possible etiological factors, such as microtrauma of the airway dehydrated epithelium and especially of the small airways resulting in maximizing epithelium dehydration [25]. The small airway epithelium seems to be the most affected by injury and repair, as shown in mice investigations [7].

At rest, the bronchial epithelium is exposed to forces such as the shear stress caused by airflow and transepithelial pressure gradient. These forces increase when VE increases. While during calm breathing the pressure gradient is -8.5 cm Hg, during exercise it can reach -20 cm Hg [34]. In vitro, high pressures have been shown to cause injury to epithelial cells and disruption of the epithelial cells layer [31]. The repeated damage and repair that occurs, can cause bronchial hyperreactivity and airway remodeling [35,36]. The increased airway inflammatory cells in elite athletes represent physical damage resulting in intense hyperpnoea that heals with rest and may not be the initial factor implying detrimental effects on the health of the respiratory system [7]. The quality of inspirated air is dependent on the type of training (e.g., indoor/outdoor) and sport-specific environments (e.g., winter and water sports) [7]. It has been proposed that exposure to allergens in the presence of microvascular leak and exudation of plasma that is caused by epithelial damage triggered by increased ventilation during physical activity, may induce airway sensitization. This sensitization could take place in non-asthmatic athletes who are atopic because they are likely to have more mast cells and more mediators [7].

In recent years, special emphasis has been given to the cellular mechanisms, which are responsible for the development of EIB. The finding of an increased number of basophilic cells in the sputum of elite athletes implicates these cells in the pathophysiology of EIB [35,37]. Many elite athletes have an increased number of neutrophils in their sputum, a finding that is consistent with airway inflammation [38,39]. The irritation of epithelial cells due to hyperosmotic environment and airway cooling leads to an increase in interleukin 8 (IL-8) production, which may be responsible for the increased neutrophilic count in the sputum of elite athletes [40]. Also, further exposure to extremely small particles and droplets, such as air pollutants, is an additional stimulus for the increased accumulation of neutrophils in the airway of these athletes [41]. The inflammation of airways caused by the presence of neutrophils may increase their sensitivity, thus contributing to the increased incidence of EIB [42,43]. The number of epithelial cells in the sputum is related to the severity of EIB and it may be an indicator of epithelial damage [25]. The finding of elevated levels of tenascin C, a glycoprotein associated with wound healing in the basal membrane of the airway endothelium of elite athletes, reinforces the view that there is a bronchial epithelium injury and its subsequent repair [36]. The bronchial epithelium injury exposes the sensory nerve ending to the inhaled microparticles and droplets, but also endogenous inflammatory mediators. This increases the contractional response of the airway to the various stimuli [43].

The concentration of leukotrienes (LTs) is increased in the bronchoalveolar lavage (BAL) of asthmatic patients, and it has been found that levels of leukotrienes E4 (LTE4) increase after exercise and especially in healthy individuals that exercise at the same intensity as elite athletes [42]. Studies with LTs receptors antagonists have shown LTs are the ones that sustain bronchial stenosis after exercise and that EIB resolves faster or is less severe when LTs receptor antagonists have been used [43,44,45].

It has not been sufficiently studied whether, in some elite athletes, airway injury remains out of the exercise period or even after the athlete is permanently removed from activity, and whether this can occur regardless of the presence or absence of EIB. In 2015, a study by Giacco et al. reported that EIB does not occur when the athletes are out of training or after the end of their athletic career [35].

To summarize, the final model results from the interaction of all aforementioned factors. The continual heavy ventilation during physical activity in elite athletes acts as early local airway damage stimulating and maintaining bronchial inflammation and delaying bronchial restoration. Inflammation is a nonspecific answer of airways to damage, which leads to repair and restoration of the normal structure and function. The process of restoration includes plasma exudation and cell transportation into the airway, a process repeated many times during a period of preparation in elite athletes. The exposure of the airway to plasma and cells over time leads to a change in the contractility of the smooth muscle that makes them more prone to bronchospasm. The bronchial reaction is a result of both those structural airway modifications, the so-called airway remodeling, and increased bronchial tonus related to higher parasympathetic activity. These changes continue with repetitive exercise periods but are also stimulated by environmental exposure where the sport is being performed. More investigations are necessary to take safe conclusions in this direction. Also, the current knowledge of the natural history of EIB in elite athletes is deficient, and more investigations are needed to identify how many athletes continue to experience EIB in the years after terminating intensive endurance exercise.

Clinical manifestation of EIB

Exercise-induced bronchospasm occurs during exercise and at six to eight minutes of intense aerobic exercise ( > 80% VO2 maximal oxygen consumption predicted). If the exercise period is shorter, symptoms may develop three to 15 minutes after completion of exercise [46]. The symptoms of EIB are like those of asthma and include sudden onset of shortness of breath, coughing, mucus production, chest pain or tightness, and wheezing that lasts between 30 to 90 minutes and usually resolves spontaneously or may be atypical and difficult to recognize, such as deficient performance and poor fitness. Some athletes also may report throat and gastrointestinal disorders [44,45]. In elite athletes, EIB may be completely asymptomatic and only recognized by specific functional tests [45,46,47]. Also, the symptoms may be different from person to person [24].

Diagnostic methods of EIB in athletes

The diagnosis of EIB in elite athletes begins with obtaining a medical history and searching for compatible symptoms during exercise and ends with the documentation of the changes in specific pulmonary function (Table 1). Athletes presenting with typical symptoms of EIB must first undergo spirometry with bronchodilator reversibility testing. The administering of bronchodilator is significant as athletes usually have higher baseline forced expiratory volume in one second (FEV1) than non-athletes, and spirometry without bronchodilator reversibility test may appear without abnormalities. Athletes in high-ventilation activities (track, soccer, ice hockey, field hockey, swimming, skiing) are more likely to have EIB symptoms compared with those in low-ventilation activities (golf, baseball, bowling, diving, weightlifting, volleyball, football) [5]. Minimal risk sports are ones in which training lasts less than five to eight minutes [5].

**Table 1:** Diagnostic tests for EIB in elite athletes EIB: Exercise-induced bronchospasm, FEV1: Forced expiratory volume in one second

Test	Criteria (FEV_1_)
Bronchodilation test	↑ FEV_1 _> 12% and 200 ml
Eucapnic voluntary hyperventilation	↓ FEV_1_ > 10%
Exercise test	↓ FEV_1_ > 10%
Methacholine test	↓ FEV_1_ > 20%
Hyperosmolar test (saline, mannitol)	↓ FEV_1_ > 15%

In elite athletes with EIB, the diagnosis is based on specific tests that estimate bronchial hyperreactivity in conditions of intense exercise as seen above in Table 1. Ideally, the athletes should be examined in the exact conditions of the exercise in which their respiratory symptoms appear. As this is usually not possible, this test can be performed with various diagnostic methods. The evaluated index is the FEV1, which is evaluated before and after the test. A reduction of FEV1 relative to pre-exercise levels of 10% at least in one of the measurements to be performed within 30 minutes of the completion of the test makes the diagnosis of EIB. The severity of EIB can be classified as mild, moderate, or severe if the rate of reduction in FEV1 from pre-exercise levels is ≥10% but <25%, >25% but <50%, or >50%, respectively. The use of alternative estimation indexes has also been considered, such as a decrease in mid-expiratory flow (MEF) ≥20% [22,25] (Table 2).

**Table 2:** Levels of EIB severity EIB: Exercise-induced bronchospasm, FEV1: Forced expiratory volume in one second

Grade	FEV_1_ decrease
Mild	>10% but <25%
Moderate	>25% but <50%
Severe	>50%

**Figure 2:**
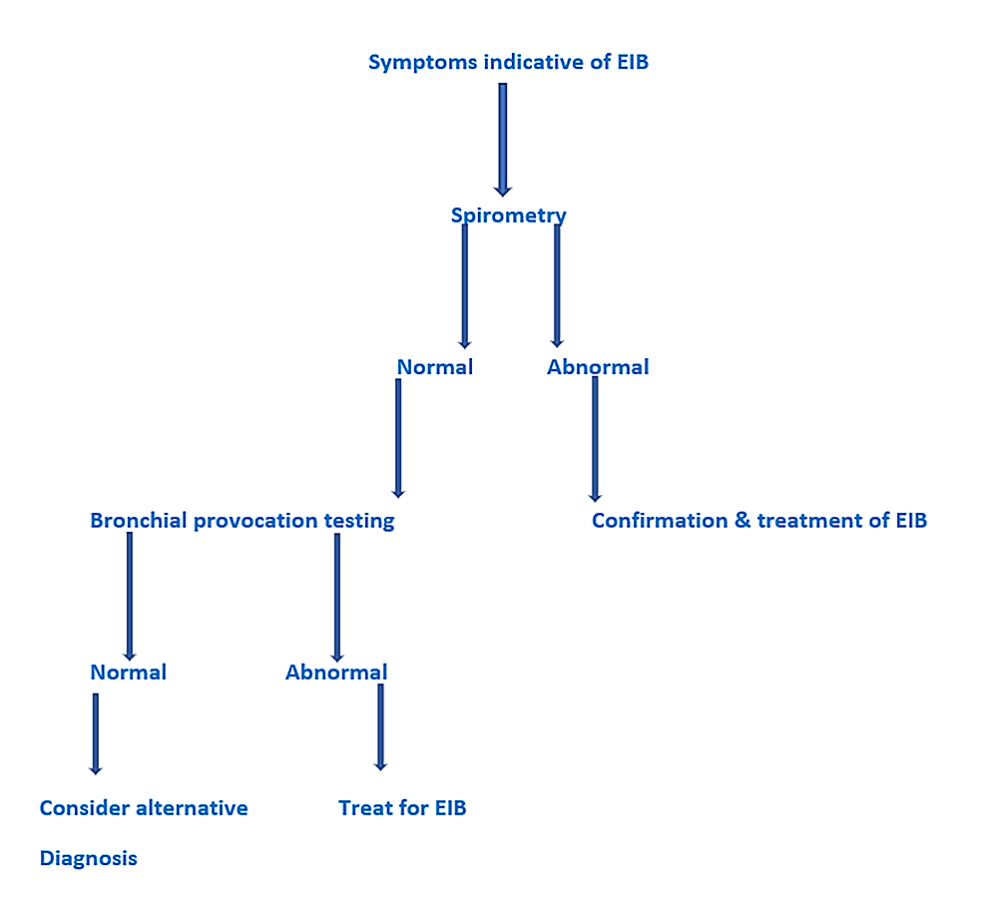
Diagnostic algorithm of EIB in elite athletes EIB: Exercise-induced bronchospasm

To date, there is no ideal test for the documentation of EIB diagnosis and various methods have been used (Figure 2).

Spirometry Flow Volume

The diagnosis of EIB can be made only when specific symptoms are associated with actual results of variable airway obstruction through either spirometry with bronchodilator reversibility examination or provocation tests. The spirometry before and after bronchodilation is the first diagnostic step for the diagnosis of EIB, and athletes presenting with characteristic symptoms should undergo this test. Elite athletes commonly have higher baseline FEV1 than nonathletes, and most elite athletes with EIB have normal spirometry at rest [48,49]. For this reason, bronchodilator administration is important to perform as spirometry without bronchodilator reversibility testing may be without abnormalities. Reversibility is defined by an increase in FEV1 of more than 200 ml and more than or equal to 12% from baseline assessment after inhalation of short-acting beta-agonists (SABA) [5]. If spirometry is ambiguous or without abnormalities, but EIB is suspected, the athlete must undergo a bronchoprovocation test, which can be through direct or indirect provocation.

Provocation Tests

Provocation tests are based on the administration of factors that induce bronchospasm (histamine, methacholine, hyperosmolar aerosols). Since the pathogenesis of EIB in elite athletes might be different from that of asthma, the methacholine provocation test does not always give the possibility to identify EIB, and it has limited application in the diagnosis of EIB in elite athletes. A positive result does not confirm EIB while a negative one does not rule it out [26]. In elite athletes, the sensitivity of the methacholine provocation test does not exceed 40% to 70% [50,51]. Inhalation of the hypertonic solution is not supported by the studies in the diagnosis of EIB in elite athletes [50,51]. Inhalation of dry mannitol powder in the diagnosis of EIB is a comparable method to the test of eucapnic voluntary hyperventilation (EVH) and the cardiopulmonary exercise test (CPET). The initial dose of mannitol is 5 mg, which gradually doubles to 160 mg. One minute after each inhalation, two spirometry tests are performed, and the test is considered positive if there is a reduction of FEV1 ≥ 10% [50,51].

Eucapnic Voluntary Hyperventilation (EVH)

This test involves inhaling a mixture of dry, frigid air containing 5% carbon dioxide (CO2) and 21% oxygen (O2) with the target to achieve the 85% of maximum voluntary ventilation (MVV) of the subject. The CO2 is added to the mixture to avoid hypocapnia, which may cause bronchospasm. The hyperventilation (22 to 30 times per minute) continues for six minutes and the FEV1 is recorded at specific intervals of more than 20 minutes after the test and compared with the initial value. The EVH test has a high sensitivity for the diagnosis of EIB in elite athletes, and the medical team of the International Olympic Committee (IOC) considers this as the criterion standard provocation test for documentation of EIB in elite athletes [1,50,51]. A 10% decrease in FEV1 is suggestive of EIB during the EVH test [5]. In general, EVH is a more sensitive and specific test, but it is expensive and is not often easily available to most physicians [5].

Open Field-Based Exercise Test

The athlete performs in an environment where symptoms of EIB appear. There are questions about the sensitivity of the method, which are related to the fact that it does not provide fully controlled conditions during the test and therefore the results cannot be completely comparable [26,47,51].

Ergospirometry/Cardiopulmonary Exercise Test (CPET)

The CPET is performed on a treadmill or a cycle ergometer in laboratory conditions, or in real sports conditions with specific portable devices [1,50]. The evaluated index is the FEV1, which is assessed before and after the CPET. A 10% or greater decrease in FEV1 compared with pretest is diagnostic for EIB [5]. During the CPET, a constant work exercise protocol, lasting eight to 12 minutes, is applied. Spirometry is performed before CPET and every five minutes for 30 to 45 minutes after the test [1,8]. If there is a high level of suspicion for EIB during exercise, spirometry can be assessed at the first and second minutes after CPET [5]. In elite athletes, during CPET, a heart rate > 90% of the predicted maximum value should be reached [8], as this increases the sensitivity of the test [52]. The CPET result is related to the levels of humidity of inspirated air and for this reason, the humidity of the laboratory room should be adjusted to < 50% [52].

Differential diagnosis and comorbidities

The athlete who presents with EIB commonly experiences symptoms during or after exercise [5,24,45,46]. However, symptoms alone are insufficient to support the diagnosis of EIB because various masquerading diseases imitate this condition [5]. The differential diagnosis of EIB requires a good medical history and appropriate testing. Exercise-induced laryngeal dysfunction (EILD) as vocal cord dysfunction or paradoxical vocal fold movement, exercise-induced laryngeal prolapsus, and exercise-induced laryngomalacia can often imitate EIB symptoms. Exercise-induced laryngeal dysfunction can occur alone or coexist with EIB [5]. Gastroesophageal reflux disease (GERD) and laryngopharyngeal reflux can exacerbate with physical activity and are frequently linked to the duration and intensity of the exercise [5]. The CPET can be used to distinguish dyspnoea on exercise from EIB in an obese patient. The CPET can reveal increased oxygen consumption rather than bronchospasm [5]. Cardiovascular disorders may be associated with palpitations, dizziness, or syncope [5]. Psychological factors can also be considered, especially if objective testing does not reveal a diagnosis [5]. A summary of the differential diagnosis of EIB is contained in Table 3.

**Table 3:** Differential diagnosis of EIB in elite athletes COPD: Chronic obstructive pulmonary disease,  ILD: Interstitial lung disease, PH: Pulmonary hypertension, GERD: Gastroesophageal reflux disease, EIB: Exercise-induced bronchospasm

1. Exercise-induced laryngeal dysfunction	a) Vocal cord dysfunction, b) Laryngeal prolapsus, c) Laryngomalacia
2. Obstructive / restrictive / vascular lung diseases	a) COPD, b) Asthma, c) Bronchiectasis, d) ILD, e) PH
3. Neuromuscular diseases	
4. Cardiovascular disease	
5. GERD	
6. Overtraining syndrome	
7. Lifestyle changes (alcohol, smoking, sleep deficiency)	
8. Depression – anxiety	

Management of EIB in elite athletes

**Figure 3:**
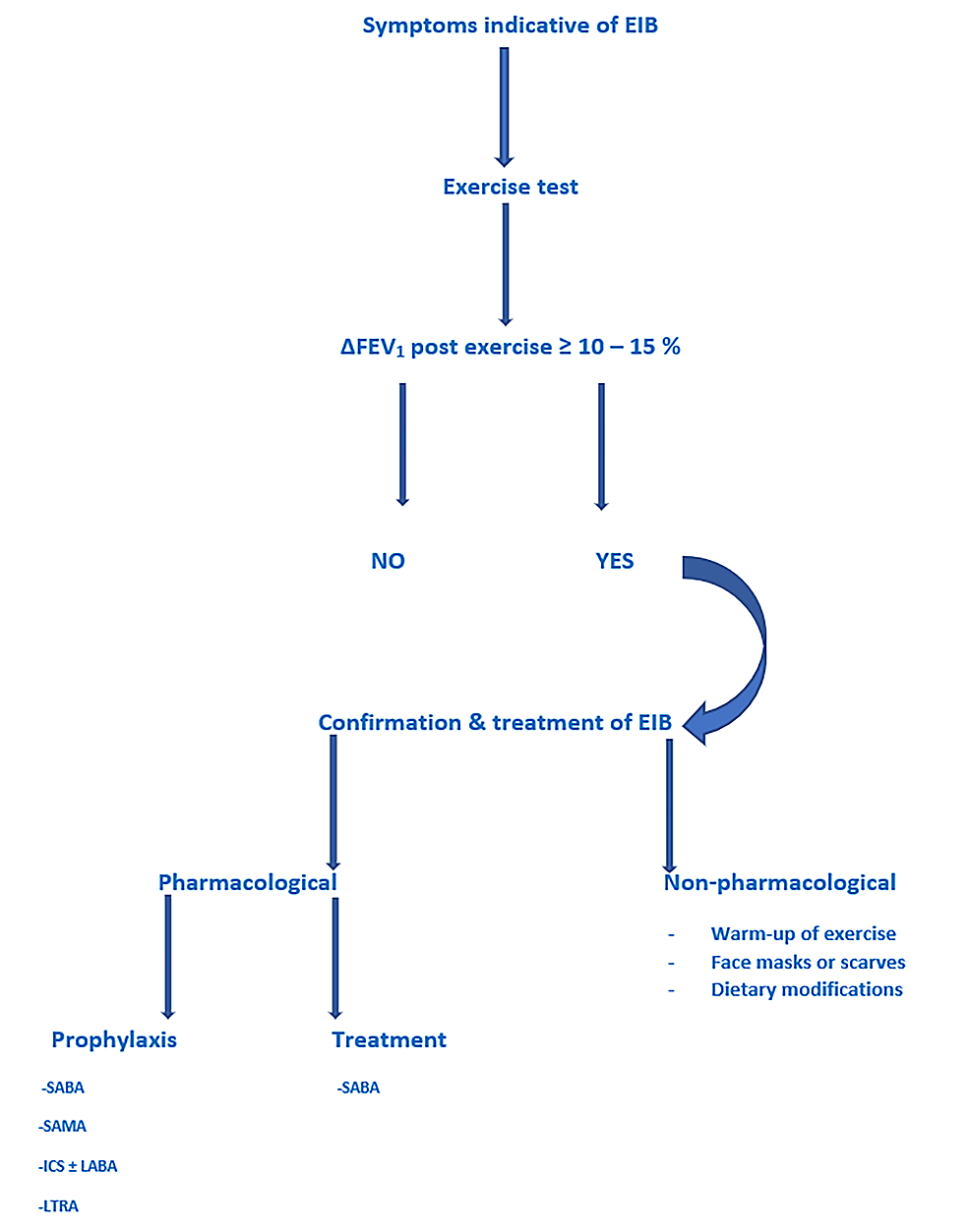
Therapeutic approach of EIB in elite athletes EIB: Exercise-induced  bronchospasm, SABA: Short-acting beta-agonists, LABA: Long-acting beta-agonist, ICS: Inhaled corticosteroids, LTRA: Leukotriene receptor antagonists, SAMA: Short-acting muscarinic antagonist

There is a high incidence of EIB in elite athletes that are underdiagnosed and undertreated [5]. The management of EIB aims to ensure the safety of the athletes during exercise, but also to help them improve their performance [53]. The treatment of EIB is divided into pharmacologic and nonpharmacologic. Once relevant tests have been conducted and diagnosis has been made, both pharmacologic and nonpharmacologic measures can be implemented to offer better control (Figure 3).

Nonpharmacologic treatment includes a pre-exercise warm-up, use of a heat exchange mask, and dietary interventions. Also, the nonpharmacologic aspect of EIB management stresses athletes’ education, reducing irritant environmental exposures as well as treating comorbidities, such as rhinitis and GERD. Warm-up includes 10 to 15 minutes of moderate-intensity exercise but is not known to be effective in elite athletes. Pre-exercise warm-up practices are often used to obtain a refractory period to lessen or avoid EIB. Common suggestions support a warm-up that includes aerobics with stretching exercises to achieve 50% to 60% of maximum heart rate [5]. Control of the environment is an alternative tactic that tries to decrease symptoms of EIB. Cold freezing air and dry environments are believed to stimulate bronchoconstriction. Mechanical barriers include face masks that feature a heat and moisture exchanger, promote humidification, and prevent water loss and diminish inhalation of particulate air pollution. However, their use does not appear to be effective in preventing EIB in elite athletes. Also, it is not easy to use them during exercise [5]. In addition, athletes must stay away from running next to busy roads or must plan outdoor exercise around low-traffic hours [5]. Many investigations have studied the effects of nutrition on EIB. Dietary changes have been shown to reduce the manifestation and severity of EIB in elite athletes. The most important nutritional intervention is the intake of fish oil rich in omega-3 fatty acids, whose anti-inflammatory action is due to antagonistic action to arachidonic acid in the induction of inflammatory mediators. The derivatives of arachidonic acid are the leukotrienes, which are a potent agent of neutrophils chemotaxis and cause strong contraction of smooth muscle fibers and bronchospasm [51]. However, the evidence supporting these changes is weak [5]. Less well studied are the effects of caffeine, which is an antagonist of adenosine receptors that can have a bronchodilator effect. The usual amounts of caffeine that have a bronchodilator result include 5 to 10 mg/kg of body weight daily (two to four cups of coffee) [5].

Pharmacologic therapy is the most effective treatment of EIB (Table 4). Elite athletes can be treated with therapies improving airway inflammation and hyperresponsiveness. The pharmacologic aspect of EIB management focuses on the use of correct medication for prevention, control of symptoms, and rescue. Athletes should be provided education on proper inhaler use and the importance of medication adherence [5,53]. Several drugs are used in EIB such as SABA, long-acting muscarinic antagonist (LAMAs), leukotriene receptor antagonists (LTRAs), and inhaled corticosteroids (ICSs). The SABAs are the first-line treatment, and they aim both to prevent EIB and treat its symptoms. They are administrated 10 to 15 minutes before exercise and protect the athlete for about two to four hours. Medications of this group stimulate the beta-2 receptors of airways and cause bronchodilation. Also, they prevent the degranulation of mast cells. Chronic or recurrent use may lead to a loss of efficacy and duration of effect. Tolerance develops due to the downregulation of beta-2 receptors on the smooth muscle in the bronchial tree and on mast cells [5,53]. The long-acting beta-agonists (LABAs) have a longer period of action but are not advised to be prescribed as monotherapy. They are used in combination with ICSs and are effective in treating EIB in elite athletes. As with SABAs, tolerance can also develop with LABAs. The LTRAs have been shown to have a significant benefit in the treatment of EIB and can be used for regular daily or intermittent administration one to two hours before exercise. The montelukast has an onset of protection against EIB within two hours following a single oral dose and its protective action lasts for up to 24 hours. They cannot reverse the acute bronchial obstruction but have not been proven to induce tolerance [5,53]. Finally, mast cells stabilizers (MCS), such as cromolyn sodium and nedocromil sodium attenuate EIB when given before exercise but have a short duration of action. Short-acting muscarinic receptors antagonists (SAMAs) are anticholinergic agents, such as ipratropium, with action on smooth muscle receptors, which leads to muscle relaxation and bronchodilation, but there is conflicting data on its impact on EIB [5]. Methylxanthines (theophylline) and antihistamines should be used with caution or selectively because they have conflicting results. They are not used as a first-line medication for the treatment of EIB in elite athletes [53]. In addition, other medication that has been studied in the treatment of EIB includes calcium channel blockers and a-adrenergic receptors antagonists, but these also have conflicting results [5,53].

**Table 4:** Pharmacologic agents for EIB treatment in elite athletes EIB: Exercise-induced bronchospasm, SABA: Short-acting beta-agonists, LABA: Long-acting beta-agonist, ICS: Inhaled corticosteroids, LTRA: Leukotriene receptor antagonists, SAMA: Short-acting muscarinic antagonist, MCS: Mast cell stabilizers

Medication	Administration
SABA	Before exercise
LABA	Recommended against daily use as a single therapy
ICS	Recommended daily use / Recommended against use only before exercise
LTRA	Recommended daily use or before exercise
SAMA	Before exercise / Conflicting data
Methylxanthines (theophylline)	Conflicting data
Antihistamines	Conflicting data
MCS	Before exercise

Discussion 

Exercise-induced bronchospasm is often observed in elite athletes whose medical history and symptomatology have neither sensitivity nor specificity for diagnosis documentation. Due to this, there is the suggestion that elite athletes be screened for EIB [54]. A negative result does not ensure long-term normal pulmonary function. It has been shown that EIB may also occur in athletes who initially had normal pulmonary function tests (PFTs) [28].

The optimization of athletic performance and safety during exercise are important reasons to evaluate all elite athletes. The high percentage of elite athletes who develop respiratory disorders during their careers should also be considered at this point [55]. To date, there have been no reports on the progression of asymptomatic EIB and whether it may have long-term effects on the pulmonary function of these athletes. Also, the role of mid-expiratory flow (MEF) or forced expiratory flow (FEF) at 25% to 75% of forced vital capacity (FVC) has not been studied in the evaluation of EIB nor the evaluation of a possible permanent small airway disease in elite athletes.

Proper hydration contributes to an athlete’s optimal performance and has been widely studied [54]. Sports with prolonged aerobic exercise have been associated with increased fluid needs. Losses of more than 2% of body weight indicate an increased risk of dehydration with serious effects on exercise capacity [55]. To date, the role of pre-exercise hydration in EIB is not known and more studies should be performed in elite athletes to highlight the role of hydration in the severity of EIB, but also in the occurrence of other pulmonary dysfunctions, such as small airway disease.

## Conclusions

Exercise-induced bronchospasm is an extremely serious and common disorder in elite athletes. The symptoms of EIB in elite athletes are nonspecific and the diagnosis needs to be confirmed with detailed and specific tests. The differential diagnosis of EIB is extensive and except for appropriate testing, it requires a good clinical history. To decrease the risk of occurrence of this disorder, athletes should be encouraged to avoid certain environmental and ambient triggers and address the frequency and intensity of their training. The SABAs are the initial therapy, but LTRAs or ICSs with or without LABAs may also be required. More studies are needed to establish whether an early diagnosis of EIB can improve the health and performance of athletes. Finally, it is necessary to examine the role of hydration before exercise in the manifestation and severity of EIB in elite athletes. 
